# Examining fertility preferences and spousal communication on contraceptive uptake among postpartum mothers in selected facilities in Abuja, Nigeria

**DOI:** 10.1186/s40834-026-00428-0

**Published:** 2026-01-27

**Authors:** Adaobi C. Iluno, Adeyemi O. Adekunle, Imose Itua

**Affiliations:** 1 Faculty of Health Studies, University of Bradford, Bradford, UK; 2 College of Medicine, University of Ibadan, Ibadan, Nigeria

**Keywords:** Family planning, Fertility preference, Contraceptive, Postpartum mothers

## Abstract

**Background:**

The postpartum period is linked to a high risk of unintended pregnancies, contributing to the high fertility rate in Nigeria. The study investigated the fertility preferences and spousal communication on contraceptives among postpartum mothers attending maternal and child health clinics in the Abuja district hospitals.

**Methods:**

A descriptive cross-sectional study was conducted, and 248 postpartum mothers were purposely selected from a cluster of women attending a postnatal clinic across four district hospitals in Abuja, Nigeria. The data were summarised using mean, standard deviation, frequency, and percentages. Bivariate analysis was conducted using the Pearson χ^2^ test at a 95% level of statistical significance.

**Results:**

Regarding the respondents’ fertility preferences, about 80% desire to have another child; however, only a few desire to have a child soon, while over 70% want a child later. This desire to have another child increases as the parity level decreases. The study revealed a strong desire to use a contraceptive method (56.5%), however, only 15.3% of the respondents were current users. The data showed a significant association (*P* = 0.0001) between frequent spousal communication and contraceptive uptake.

**Conclusion:**

The study revealed a high unmet need for spacing, a strong desire for contraceptives and a low uptake among postpartum mothers. Improving access to family planning (FP) methods and including spousal communication in FP interventions can help postpartum mothers fulfil fertility preferences, reducing unintended pregnancies and negative reproductive health issues.

## Introduction

Studies have reported that mothers across the developing world would like to space or limit the number of children they have, yet non-use of contraceptives is substantially high among them despite their sexual exposure [1, 2, 3]. About 218 million mothers of reproductive age, 15–49, in developing countries have an unmet need for modern contraception [4]. Serving all mothers in developing countries who currently have an unmet need for modern contraceptives would reduce unintended pregnancies by 68%, unsafe abortions by 72% and maternal deaths by 62% [4].

According to the National Demographic and Health Survey (NDHS) 2023-24 data, Nigeria has a 20% contraceptive prevalence rate among married mothers aged 15 to 49 years, with only 15% of these as users of modern contraceptive methods [5]. There is an elevated risk of under-five mortality for children whose mothers had an unmet need for spacing or limiting childbearing than those whose mothers had met needs [1]. Investing in contraceptives in Nigeria is paramount to reducing global health inequities, which remain a cornerstone of the Sustainable Development Goal (SDG) agenda.

Family planning can help meet the reproductive goals of postpartum mothers who wish to initiate and use contraceptives during the first year after delivery. The postpartum period is a time with a high risk of unwanted pregnancies [6], and this has been one of the contributing factors to the high fertility rate in Nigeria. Evidence has shown that by 6 months post-delivery, most mothers have resumed sexual activity and are yet to adopt family planning [7]. Barriers to contraceptive uptake by postpartum mothers include misconceptions about pregnancy risk, concerns about health consequences and side effects, pressure to become pregnant, and opposition from family members [8, 9].

Knowledge and awareness of family planning methods in Nigeria are considerably high [10], yet uptake of family planning among postpartum mothers has remained low [5]. Borda and Winfrey reported a high unmet need for family planning in their study among postpartum mothers [11]. This means that postpartum mothers are more susceptible to unintended pregnancy and are likely to embrace contraceptive methods if appropriately targeted. Unfortunately, the fertility preferences and spousal communication on contraceptive uptake among this group in Nigeria have not been suitably documented, thus limiting the opportunity to develop bespoke family planning programs.

Fertility preferences in African countries have shown that women have a high desire for more children [12, 13, 14]. This means that despite modernisation and changes in lifestyle, many mothers in African countries still have large families. The fertility rate in Nigeria has slowly decreased over the past three decades. In 1990, the average Nigerian woman had 6.0 children. By 2018, this number had only decreased to 5.3 children per woman, and the latest data from the NDHS (2023–2024) reports the fertility rate as 4.8 [5]. This reflects a gradual decline in the fertility rate in Nigeria, despite the increased family planning interventions that have been implemented. Continued efforts are necessary to achieve even greater reductions in the future.

In Sub-Saharan Africa, most family planning interventions have traditionally focused on women, despite the significant role men often play in these contexts [15]. Actively including men as key family planning clients can promote more collaborative decision-making regarding contraceptive use [15, 16, 17]. Joint decisions about contraception by men and women could enhance family planning efforts and increase the utilisation of reproductive health services [18]. Spousal communication is essential for family planning uptake, as it enables discussions about beliefs, addresses uncertainties, and facilitates decision-making [19].

Studies have explored the unmet need for contraceptives among mothers and the different factors that influence it [20, 21]. Still, a few have examined fertility preferences among postpartum mothers and spousal communication on contraceptive uptake among them. This study, therefore, examined the fertility preferences and spousal communication on contraceptive uptake among postpartum mothers attending maternal and child health clinics within the Federal Capital Territory (FCT) district hospitals in Abuja, Nigeria.

## Methods

A descriptive cross-sectional study design was used for the study. The study was conducted at four district hospitals in major cities in the Federal Capital Territory, Abuja, the capital city of Nigeria. The study population consisted of postpartum mothers utilising the maternal and child health clinic for any purpose and were willing to provide informed consent (both verbal and written) to participate. Postpartum mothers in this study refer to mothers in the first 12 months following their last childbirth. The sample size was determined using Lwanga and Lemeshow’s formula [22]. The desired confidence level was set at 95%, and the prevalence of the studied attribute (unmet need for family planning in the study population for the parent study) was 0.103 (Doctor et al., 2013). Additionally, a precision level of 4% was chosen to ensure higher accuracy and an increased sample size. The calculated minimum sample size was 222 with a non-response rate adjustment calculated as 222 * 1/ (1 – NR), where NR is the non-response rate (10%) = 25. This was added to the calculated minimum sample size to make a total of 247. A rounded sample size of 248 was used to distribute participants evenly across the 4 selected healthcare facilities. The instrument was developed based on a comprehensive literature review and the study’s objectives. To ensure its reliability, it was designed, reviewed, and refined by the authors and then pre-tested among 20 postpartum mothers attending maternal and child health clinics at the city centre of Ibadan, Nigeria. The location of the clinics used shares the same characteristics as the location of the district hospital in Abuja city. A template was designed on SPSS (version 20) for entering the coded data. The reliability coefficient of the questionnaire was determined using the Cronbach’s Alpha model technique of SPSS (version 20). In this approach, a reliability coefficient not less than 0.5 was used to adjudge the questionnaire as being reliable. The pre-test showed a reliability coefficient of 0.975, implying that the instrument was reliable. Revisions, such as adjustment of structural layout, clear labelling of sections and simplifying some instructions, were made to the instrument before it was finally used. To ensure data quality, trained field assistants administered the questionnaires using a standardised guide developed after pre-testing. The guide provided uniform explanations, ensuring that any clarifications offered in the field were consistent and did not introduce interviewer bias. Purposive sampling with consecutive recruitment was used because the clinics operated on a walk-in basis. Eligible postpartum women were therefore approached consecutively as they arrived for postnatal visits. Sixty-two postpartum mothers were selected as they came into the clinic in consecutive order for the interview in each of the FCT District Hospitals, Abuja. Consent of the participant was sought before the administration of the questionnaire. There was an explanation of the purpose of the research, the time that would be spent, and the benefits of the research. The questionnaires were collated immediately after each clinic visit, and the field assistant cross-checked them for completeness.

The data were summarised using the mean, standard deviation, frequency and percentages. Bivariate analysis was conducted using the Pearson *χ*^2^ test, and factors associated with the outcome were determined using binary logistic regression analysis at a 95% level of statistical significance. The results of the analysed data were presented using tables and figures.

## Results

### Socio-demographic characteristics of respondents

There was an equal number (62) of respondents represented across all the hospitals in all the locations used (Maitama General Hospital, Gwarinpa General Hospital, Wuse General Hospital, and Asokoro General Hospital). The age of the respondents ranged between 19 and 42 years, with a mean age of 29.8 ± 4.3 years. A greater percentage of the respondents were within the age groups of 25–34 years (75.8%), while others were within the age group 15–24 years (10.5%) and 35–44 years (13.7%). Most (99.6%) of the postpartum mothers were married, and only 0.4% cohabited. More than half (57.3%) of the respondents have attended tertiary education, while 42.3% have secondary education and 0.4% have primary education.

### Respondents’ awareness of family planning

Table 1 shows the number of respondents who have heard of family planning. Among all the reported sources of information on family planning, health clinics (98.5%), health workers (97.2%), friends (43.1%), radio (15.3%), relatives (14.1%) and television (14.1%) were the highest sources of information. The workplace (5.2%), church (4.4%), internet (2%) and conferences (0.8%) were not as useful in disseminating information about family planning. When asked about the type of modern methods they know, condoms (100%), injectables (61.7%), birth control pills (55.6%), implants (50.6%), and IUD (43.1%) were the most mentioned.

**Table 1:** Respondents’ level of awareness towards family planning

*N* = 248		
Awareness-related variables	Yes (%)	No (%)
*Ever heard of Family Planning*	248 (100)	0 (0)
*Source of information*		
Health clinic	245 (98.8)	3 (1.2)
Health worker	241 (97.2)	7 (2.8)
Family	107 (43.1)	141 (56.9)
Radio	38 (15.3)	210 (84.7)
Relatives	35 (14.1)	213 (85.9)
Television	35 (14.1)	213 (85.9)
Workplace	13 (5.2)	235 (94.8)
Church	11 (4.4)	237 (95.6)
Internet	5 (2)	243 (98)
Newspaper	5 (2)	243 (98)
Conferences	2 (0.8)	246 (99.2)
*Type of modern Family planning*		
*method aware of*		
Condom	248 (100)	0 (0)
Injectables	153 (61.7)	95 (38.3)
Birth control pills	138 (55.6)	110 (44.4)
Implants	126 (50.8)	122 (49.2)
IUD	107 (43.1)	141 (56.9)
Cervical cap	10 (4)	238 (96)
Female sterilization	9 (3.6)	239 (96.4)
Vasectomy	8 (3.2)	240 (96.8)
Vaginal ring	6 (2.4)	242 (97.6)
Patch	3 (1.2)	245 (98.8)

### Fertility preferences of respondents

As shown in Table 2, most postpartum mothers were within the 7-week to 3-month postpartum period (39.9%) and the 7-month to 9-month postpartum period (30.2%). The findings revealed that 38.7% of the respondents had only one child, while 28.6% and 20.2% had two and three children, respectively. Only 7.3% and 5.2% had four or more children, respectively. More than 80% expressed a desire to have another child, whereas 19% indicated they do not wish to have another child. Seven out of ten (7%) postpartum mothers who wish to have more children prefer to wait, 6% want it soon, and 2% were undecided about the timing of having another child.

**Fig. 1:**
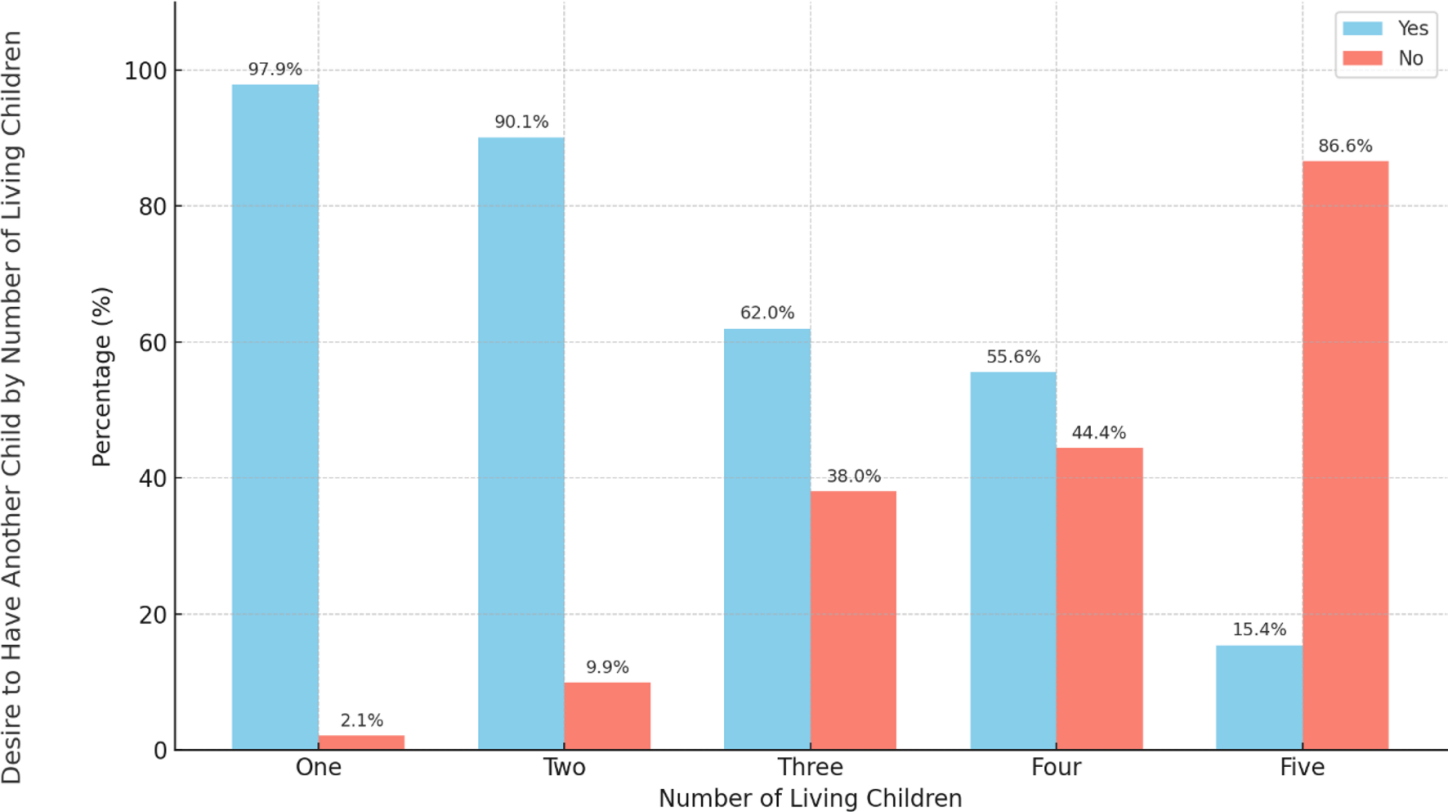
Comparison of number of living children with their desire to have another child

Figure 1 shows a decrease in the percentage of mothers who desire to have another child with an increase in the respondents’ parity level. The desire was highest (97.9%) among mothers with one living child and lowest (15.4%) among mothers with more than four living children.

**Table 2:** Respondents’ fertility preferences

Fertility Preference	*N*	%
*No of living children (N = 248)*		
One	96	38.7
Two	71	28.6
Three	50	20.2
Four	18	7.3
More than 4	13	5.2
*Postpartum period (N = 248)*		
7weeks – 3months	99	39.9
7–9 months	75	30.2
1–6 weeks	55	21
4–6 months	17	6.9
10–12 months	5	2
*Desire to have another child (N = 248)*		
Yes	201	81
No	47	19
*Additional number of children desired to*		
*have (N = 248)*		
Two	84	33.9
One	71	28.5
None	47	19
Three	27	10.9
More than 3	19	7.7
*Timing of next birth (N = 248)*		
Will want to have another later (after two	176	71
years of delivery)		
I want no more	47	19
Will want to have another soon (within one	15	6
year of delivery)		
Will want to have another but have not	5	2
decided when to		
I am undecided about having another	5	2
*Perceived ideal number of children (N = 248)*		
Four	121	48.8
Three	88	35.5
More than 4	34	13.7
Two	5	2

### The pattern of utilisation of contraceptives among respondents

More than half (56.5%) of the respondents expressed their desire to use contraceptives, while only 15.3% are currently using contraceptives. When respondents who do not desire to use a family planning method were asked the reasons for their lack of desire, most (35.2%) mentioned its “side effects”, while 31.5% said, “they don’t think it is necessary”. Other reasons mentioned included “lack of husband’s approval” 11.1%, “future use after birth 10%”, and “religion” 3.7%. More details are shown in Table 3.

**Fig. 2:**
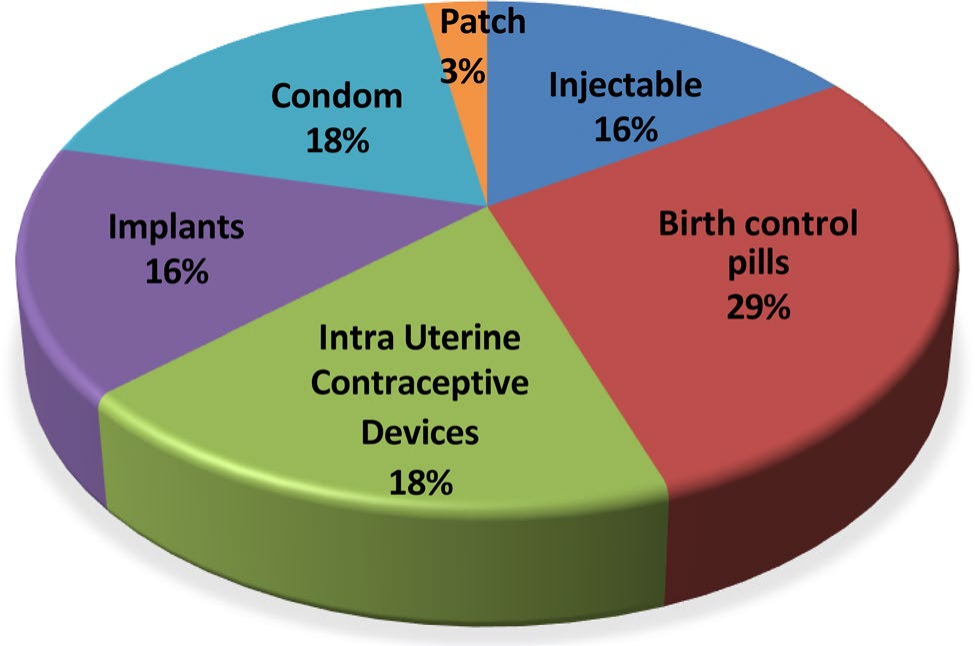
Types of modern contraceptives used by respondents

Birth control pills were the most used modern contraceptive at 28.9%, followed by Intra Uterine Contraceptive Devices (IUCD), 18.4% and condoms, 18.4%. The method that was least mentioned was the patch 0.4%. See Fig. 2.

**Table 3:** Respondents’ pattern of utilisation of a modern contraceptive

Pattern of utilisation of modern family planning methods	*N*	%
*Desire to use any modern contraceptive* *N* = 248		
Yes	140	56.5
No	103	40.7
Undecided	5	2.8
*Current use of any modern contraceptive* *N* = 248		
No	210	84.7
Yes	38	15.3
Respondent’s reasons for not desiring to use a contraceptive method* n = 108		
It has side effects	38	35.2
I do not think it is necessary	34	31.5
I do not have my husband’s approval	12	11.2
Will use after childbirth	11	10.3
My religion does not approve of it	4	3.7
My husband travelled	2	1.8
I had a delay before childbirth, and do not want to delay it again	2	1.8
I know how to control myself	2	1.8
Children are from God and should not be controlled	1	0.9
Health reasons	1	0.9
Inadequate information on family planning	1	0.9

*open responses

### Current use of contraceptives among respondents by some social demographic characteristics

Table 4 shows that 13.7% of postpartum mothers currently using a modern family planning method are aged 25–34, while the least (0.4%) are in the 15–24-year age group. Modern family planning usage is highest (10.5%) among postpartum mothers with tertiary education. Among the 84.7% of postpartum mothers not currently using any modern family planning, 34.5% are within the postpartum period of 7 weeks to 3 months, and 22.6% are in the 7 to 9 months range. Use of modern family planning is highest among postpartum mothers with two (4.4%) and three (5.2%) children. 36.3% of postpartum mothers with only one child are not currently using a modern family planning method.

**Table 4:** Current use of any modern family planning method among respondents by Age, level of education, postpartum period, location of health clinic and number of living children

Socio-demographic characteristics		Current use of the modern family planning method (*N* = 248)	
		Yes (%)	No (%)
*Age at last birthday in years*	25–34	34 (13.7)	154 (62.1)
	35–44	3 (1.2)	31 (12.5)
	15–24	1 (0.4)	25 (10.1)
*Level of education*	Tertiary	26 (10.5)	116 (46.8)
	Secondary	12 (4.8)	93 (37.5)
	Primary	0 (0)	1 (0.4)
*Postpartum period*	7-9months	19 (7.7)	56 (22.6)
	7 weeks − 3 months	14 (5.6)	85 (34.3)
	4-6months	3 (1.2)	14 (5.6)
	1–6 weeks	1 (0.4)	51 (20.6)
	10-12months	1 (0.4)	4 (1.6)
*No of living children*	Three	13 (5.2)	37 (14.9)
	Two	11 (4.4)	60 (24.2)
	One	6 (2.4)	90 (36.3)
	Four	6 (2.4)	12 (4.8)
	More than 4	2 (0.8)	11 (4.4)

### Spousal communication among respondents

Majority (71%) of respondents have ever discussed family planning with their spouses, while about 29% have never discussed it with their spouses. Additionally, 63.7% consistently discuss family planning methods with their spouses (See Table 5a).

Spousal communication about modern family planning methods among respondents was categorised into frequent and infrequent spousal communication. Frequent spousal communication refers to women who have ever discussed family planning with their spouse frequently. In contrast, infrequent spousal communication refers to those who have never discussed family planning with their spouses or rarely discuss it. The majority (63.7%) of the respondents had frequent spousal communication, while 36.3% had infrequent spousal communication. Details are provided in Table 5b below.

**Table 5:** a-b spousal communication on family planning among respondents

**a) Spousal Communication**	***N***	**%**
*Ever discussed Family* *Planning with spouse N = 248*		
Yes	176	71
No	72	29
*Frequency of spousal* *Communication N = 248*		
Frequently Never	158 72	64 29
Rarely	18	7
**b)** **Categories of spousal communication** ***N* = 248**	**N**	**%**
Infrequent	90	36
Frequent	158	64

### Association between spousal communication and family planning use

Based on Pearson’s Chi-squared test, there is a significant association between spousal communication and contraceptive use (χ2 = 15.65, *p-value* = 0.0001, df = 1). Mothers with frequent spousal communication were more likely to use family planning methods compared to those with infrequent communication. Further results from the Yule coefficient indicate a strong positive association (Q = 0.784). The odds ratio of 8.25 (95% CI: 2.46–27.66) shows that the odds of using family planning are almost eight times higher among couples with frequent spousal communication compared to those with infrequent spousal communication. Since the 95% confidence interval value of the Odds Ratio is greater than one, the association is statistically significant and not likely to have occurred by chance. More details are provided in Table 6 below.

**Table 6:** Association between spousal communication and family planning use

Family Planning Use (*N* = 248)					
**Spousal communication**		Yes (%)	No (%)	Total (%)	
	Infrequent	3(3.3)	87(96.7)	90(100)	
	Frequent	35(22.2)	123(77.8)	158(100)	
	Total (%)	38(15.3)	210(84.7)	248(100)	

Observed X^2^ = 15.65 Critical X^2^ = 3.84 *P* = 0.0001 df = 1 Q of Yule = 0.784 OR = 8.25 CI (95%) = [2.46–27.66]

The study found that while awareness of family planning was universal among postpartum mothers, actual contraceptive use was low at 15.3%, despite 56.5% expressing a desire to use it. Fertility preferences showed that the desire for more children declined as the number of living children increased. Most women cited fear of side effects, lack of perceived need, and husband’s disapproval as barriers. Importantly, women who had supportive and open communication with their spouses were significantly more likely to use contraceptive methods.

## Discussion

The study explored fertility preferences and spousal communication on contraceptives among postpartum mothers attending maternal and child health clinics in four district hospitals in Abuja, Nigeria.

All the respondents had heard of family planning and were aware of at least one contraceptive method, just as reported in some studies [10, 23]. However, almost half (43.1%) of the mothers reported friends as their source of information, which makes them susceptible to exposure to non-factual and inadequate information on family planning. This highlights the urgent need to strengthen accurate, evidence-based health education through trusted sources such as healthcare providers, community health workers, and media campaigns.

The study’s findings revealed that while a majority (81%) of respondents expressed a desire to have another child, only a small percentage (6%) wished to have one soon. In contrast, a larger proportion (71%) indicated a preference to have a child later. Additionally, approximately 19% of the postpartum mothers surveyed stated that they did not wish to have another child at all. When comparing this result with the 2023–2024 NDHS data, 28% of mothers reported wanting to have another child soon, while 33% wanted another later and 27% did not want any more children [5]. This shows that there is a good proportion of Nigerian postpartum mothers whose fertility option is to either delay or limit childbearing. Thus, there is a need to tailor maternal health and postpartum counselling programs to address evolving fertility preferences while offering accessible and modern family planning methods such as long-acting reversible contraceptives (LARCs) to support women in achieving their reproductive goals.

A study conducted in Ghana argued that mothers’ fertility preferences change over time for various reasons but may remain constant when their ideal family size is attained [12]. In this study, a number of mothers reported their perceived ideal number of children to be “four”, and interestingly, the study found that mothers with four living children were the least likely to desire another child. This therefore shows a possible relationship between parity level and fertility preferences. The finding that four is the ideal number of children for most mothers in this study also suggests a reduction in the desire for large families among Nigerian mothers. Over the years, the total fertility rate in Nigeria has declined from 6.0 births per woman as estimated in 1990 to 5.3 in 2018 and then to 4.8 in the latest 2023-24 NDHS [5]. This presents an opportunity for policymakers to invest in education, women’s empowerment, and reproductive health programs that can reinforce and support informed family size decisions.

The result of this study also showed that more than half of the postpartum mothers desire to use a family planning method. This shows their future intention to use a modern contraceptive, which is a good indication of future high demand and use if all the impediments hindering its uptake are removed. Although family planning services were provided at the health facilities where the study was conducted, only 15.3% were current users. Studies have reported that visiting a health clinic is strongly related to the use of contraceptives if contraceptive methods can only be obtained in a clinic [24, 25, 26]. However, this study shows otherwise. More so, a study among Ethiopian mothers confirmed that, while there is a strong association between clinic visits and contraceptive use, this association are not overly correlated [27]. Similar to another study [28], the major reasons mentioned in this study for not desiring to use a contraceptive are its side effects (35.2%) and the fact that the mothers think it is not necessary (31.5%) and this is likely to be addressed with prenatal counselling as mentioned in another study with a similar study population [29]. In this study, 11.2% of postpartum mothers cited a lack of husband’s approval as a barrier to family planning. Studies have shown that women may not take up a service without their husband’s consent [30, 31]. This underscores the strong influence of partners on unmet contraceptive needs, highlighting the need for greater male involvement in family planning programs.

Findings indicate that contraceptive use is low for mothers with only one child, peaks for those with three children, and declines for mothers with four or more. Additionally, usage is highest among postpartum mothers with tertiary education, and this is consistent with the 2023-24 NDHS, which shows that contraceptive use in Nigeria increases with educational attainment and the number of living children [5]. The findings from other studies support the explanation that educated women are more likely to use contraception because education enhances empowerment and agency, which in turn improves communication and autonomy in contraceptive decision-making [32, 33]. Many postpartum mothers in this study who do not use modern family planning methods are in the postpartum period of 7 weeks to 3 months and 7 to 9 months. This was also reported by another study that many women do not use contraception after giving birth and become pregnant again sooner than desired [34]. This shows the need for targeted interventions during these critical phases. Family planning programs could be designed to target first-time mothers, high-parity women and mothers with less educational background who are less likely to use a contraceptive.

The study revealed a strong positive association between spousal communication and contraceptive uptake. Similar to findings in other studies [19, 35, 36], women who discuss family planning frequently with their spouses are more likely to use a contraceptive than mothers who do not. This finding highlights the potential role of contraceptive spousal dialogue in promoting family planning interventions. These interventions should therefore incorporate couple-focused education and counselling strategies to encourage mutual decision-making around fertility management.

### Study limitation

The study was conducted with a small sample size within facilities based around major cities in Abuja, hence excluding mothers across other areas within the country, which may affect the generalisation of the results among postpartum mothers in Nigeria. However, scientific steps, including a 4% precision level, adjustment for non-response, and the selection of multiple health facilities, were implemented to ensure the study’s results accurately reflect the experiences of postpartum mothers. There is a need for further research which targets a more inclusive and larger population to ensure that all women are inclusively represented.

### Conclusion and recommendations

This study highlights a critical gap between awareness and actual use of modern contraceptives among postpartum mothers in Abuja. Despite universal knowledge, primarily acquired through health facilities and providers, only 15.3% of respondents reported current contraceptive use. This disparity indicates that awareness alone is insufficient to drive uptake. This calls for a more holistic approach to family planning programs that goes beyond information provision to addressing fear of side effects, myths, social norms, perceptions and gender dynamics.

Fertility preferences further influenced the contraceptive behaviour of postpartum women. While 81% of respondents desired more children, the majority preferred a two-year interval, consistent with healthy spacing guidelines. Notably, the desire for more children declined as parity increased, from 97.9% among mothers with one child to 15.4% among those with five, highlighting shifting reproductive goals and the need for responsive parity-sensitive family planning counselling that aligns with women’s evolving fertility preferences.

Importantly, spousal communication emerged as a strong predictor of contraceptive use. Mothers who frequently discussed family planning with their partners were eight times more likely to adopt contraception, emphasising the value of involving men in family planning interventions, encouraging shared decision-making and mutual support. Interventions should include male involvement initiatives to foster partner support in contraceptive uptake.

The study highlights the significance of addressing fertility preferences among mothers within the first year postpartum and the impact of spousal communication on contraceptive uptake. Therefore, targeted interventions during this period, involving both women and their partners, are essential. Enhancing support for couples’ contraceptive decision-making can help reduce unintended pregnancies, unsafe abortions, and adverse maternal and child health outcomes.

## Data Availability

The Data analysed are available upon request from the corresponding author.

## Abbreviations

FCT: Federal Capital Territory
IUCD: Intra-Uterine Contraceptive Device
SDG: Sustainable Development Goals
NDHS: National Demographic Health Survey
NPC: National Population Commission
NR: Non-response Rate
UI: University of Ibadan
UCH: University College Hospital
